# Severe intestinal malabsorption associated with ACE inhibitor or angiotensin receptor blocker treatment. An observational cohort study in Germany and Italy

**DOI:** 10.1002/pds.4402

**Published:** 2018-05-01

**Authors:** Peter Malfertheiner, Claudio Ripellino, Nazarena Cataldo

**Affiliations:** ^1^ Klinik für Gastroenterologie, Hepatologie und Infektiologie Otto‐von‐Guericke Universität Magdeburg Germany; ^2^ HEOR Consultant Freelance Milan Italy; ^3^ Quintiles IMS Milan Italy

**Keywords:** ACE‐i, ARB, coeliac disease, malabsorption, olmesartan, outcome research, pharmacoepidemiology, small intestine, sprue‐like enteropathy

## Abstract

**Purpose:**

The angiotensin II receptor blocker (ARB) olmesartan has been recently associated with sprue‐like enteropathy (SLE), a gastrointestinal condition characterized by intestinal malabsorption (IM) and severe diarrhea. Whether the increased risk of SLE is substance‐specific or a class effect involving all ARBs is uncertain. The aim of this study is to assess the risk of enteropathy associated with ARBs and angiotensin converting enzyme inhibitors (ACE‐i) by using data from large administrative and claim databases.

**Methods:**

We obtained data from Italian local health‐care units and a large German claim database and included patients treated with olmesartan, other ARBs, and ACE‐i. In the absence of a specific diagnosis code for SLE, International Classification of Diseases codes for IM were used. Analysis implemented a Poisson regression with robust error variance procedure, which allowed accounting for different clusters (local health‐care units and countries) and correctly estimating the standard error for the relative risk of rare event occurrence.

**Results:**

Patients were divided into 3 groups: olmesartan (25.591, 5.5%), other ARBs (104.901, 22.5%), and ACE‐i patients (334.951, 72.0%). Baseline characteristics were similar overall. The incidence of unspecified IM in ACE‐i patients was not different compared with that of olmesartan, whereas a higher rate ratio was observed when comparing ARB patients with the olmesartan group (RR: 2.50, 95% CI 1.21 to 5.19, P .01). When International Classification of Diseases codes for coeliac disease were included, no differences were observed.

**Conclusions:**

We could not confirm previous findings of a higher risk of malabsorption in olmesartan‐only patients, and drug‐induced enteropathy should be considered the result of exposure to the class of ARBs rather than a specific drug‐related effect.

## 1 INTRODUCTION

Clinical picture of sprue‐like enteropathy (SLE) resembles celiac disease and presents with symptoms varying from mild oligosymptomatic (anemia and irritable bowel syndrome‐like symptoms) to severe malabsorption and chronic diarrhea with substantial weight loss.^1^ Both pathologies share the characteristic histological findings of duodenal/intestinal villous atrophy.^2^, ^3^ Sprue‐like enteropathy is distinct from the “classical” gluten‐induced celiac disease as patients have a negative antibody response to gliadin, endomysium, and transglutaminase and are nonresponsive to a gluten‐free diet.^4^ Causes of SLE are heterogeneous and, among others, iatrogenic etiology is common: alcohol, antibiotics, nonsteroidal antiinflammatory agents, chemotherapeutics, immunosuppressants, and, recently, olmesartan (an angiotensin‐II receptor blocker, ARB) have been associated with SLE occurrence.^5^ The association between severe SLE and the treatment with olmesartan medoxomil (OM) has initially been reported in a case series of 22 patients diagnosed with refractory celiac disease^6^ followed by a small series of individual case reports.^4^^,^ 7, 8, 9, 10 A large observational cohort study, based on the French health insurance claim database and using International Classification of Diseases, Tenth Revision (ICD‐10) codes for intestinal malabsorption (IM) and coeliac disease diagnosis, highlighted an increased risk of hospitalization in patients treated with OM when compared with other ARBs and angiotensin converting enzyme inhibitors (ACE‐i).^11^ Otherwise, it is not appropriate to rule out the class‐effect hypothesis^12^ because recent case reports suggested an association between severe enteropathy and other ARBs, including valsartan, irbesartan, telmisartan, eprosartan, losartan, and candesartan.^10^^,^ 13, 14, 15, 16, 17, 18, 19, 20 A large population‐based study of ARB‐treated patients managed by the general practitioners in Italy and Germany showed similar low proportions of unspecified IM diagnosis among the different drugs belonging to the same class. This suggests the absence of differences among different ARBs and shows the very low incidence of IM in association with their intake.^21^ These previous findings even more emphasize the need to clarify the role of hypertensive medications in the onset of severe forms of enteropathy.

The present study aimed at assessing the risk of enteropathy associated with ARBs and ACE‐i in 2 European countries (Italy and Germany) and at assessing a potential increased risk for OM.

**Key points**

- A relatively low incidence rate of intestinal malabsorption hospitalizations in a large cohort of patients treated with ARBs or ACEIs in Italy and Germany is found.
- Previous findings about the higher risk of malabsorption linked exclusively with olmesatan‐treated patients are not confirmed.
- ARBs other than olmesartan are associated with a significantly increased risk of unclassified malabsorption compared with the olmesartan group.
- ACEi patients present with a nonsignificant lower risk of malabsorption than all ARBs users.
- Drug‐associated enteropathy should be considered as the potential consequence of the entire ARB class exposure rather than a specific drug‐related effect.

## 2 MATERIAL AND METHODS

### 2.1 Study design and population

This study was a noninterventional retrospective analysis based on longitudinal secondary data sources. Two different cohorts were constructed from administrative and claims databases in Italy and Germany, respectively. In Italy, all patients who initiated treatment with an ACE‐i (Anatomical Therapeutical Classification [ATC] codes: C09A, C09B) or an ARB (ATC codes: C09C, C09D) between January 1, 2011 and December 31, 2015 were considered. Furthermore, according to the current availability of validated data per local health‐care unit (LHU), 3 different selection periods were considered for the analysis to ensure the accuracy and efficiency of results produced (see Table S1). For German patients, the selection period went from January 1, 2011 to December 31, 2014. The first prescription of ACE‐i or ARB during this period constituted the entry date in the cohort (index date). To limit the study to treatment‐naïve patients for the studied drugs, patients who had at least 1 prescription containing ACE‐i or ARB during the 12 months preceding the index date (preselection period) were excluded from the analysis. In addition, also patients that at the index date had prescriptions falling in more than 1 of the treatment groups of interest were excluded. Further exclusion criteria applied in the preselection period were hospitalization for IM (Italy: ICD‐9 code: 579.x; Germany: K90.x), any exemption for coeliac disease (only available in Italy: code RI0060), any coeliac disease‐specific serological testing (only available in Italy: 90.53.D, 90.49.5, 90.47.E codes), and any IM diagnosis (only available in Germany: ICD‐10 codes K90.x). The 2 cohorts were then pooled into the final database used in the analysis.

### 2.2 Exposure definition

The following 3 treatment exposures were investigated: exposure to OM, exposure to other ARBs, and exposure to ACE‐i. Exposure to treatment was defined as both monotherapy and fixed combinations with diuretics and/or calcium channel blockers. The total number of days on therapy was analyzed by means of the defined daily dose. Starting from the treatment at the index date (OM, other ARB, or ACE‐i), the end of the exposure period was determined by treatment discontinuation or switch to a different antihypertensive therapy. Discontinuation was defined as any gap of more than 90 days between refilling prescriptions.

### 2.3 Outcomes

Given the lack of a specific diagnosis code defining SLE, ICD codes for IM were considered as proxies for the diagnosis of drug‐associated sprue, both for primary and secondary outcomes, as previously used in the study by Basson et al.^11^ The primary outcome was hospitalization with a discharge diagnosis of unspecified IM (Germany: ICD‐10 codes K90.4, K90.8 or K90.9; Italy: ICD‐9 codes: 579.8 or 579.9).

The secondary outcome was hospitalization with a discharge diagnosis of IM (Germany: ICD‐10 codes K90.x; Italy: ICD‐9 codes 579.x) to broaden the investigation to all ICD codes and to discern subsequently the cases among unspecified, coeliac disease and other syndromes of malabsorption. Patients were censored at the first event, death, or end of the study (Germany: December 31, 2014; Italy: December 31, 2015).

### 2.4 Data sources

#### 2.4.1 Italy

We obtained access to the administrative databases of 5 Italian LHUs: Bergamo, Toscana sud‐est—including Grosseto, Siena, and Arezzo—Roma3, Matera, and Palermo. The selected LHUs contained information on over 4 million registered patients of the 59.7 million registered inhabitants in Italy (2014 census) regarding more or less all services provided in a health‐care environment.^22^, ^23^ Each LHU supplied patient‐level data on billable claims (prescriptions from pharmacies in the area, flow of outpatient specialist examinations and of diagnostic tests and procedures, and flow of hospital discharge forms) and demographic data, retrieved from the specific databases. These sources and their integration are a powerful tool supporting conventional methods used in epidemiological studies.^24^, ^25^, ^26^, ^27^, ^28^, ^29^ Diagnoses and procedures associated with hospital utilization were recorded by using the ICD‐9 Clinical Modification, while prescription forms contained information on strength, number of tablets, and date of dispensation according to the ATC codes.

#### 2.4.2 Germany

A large claim database of people covered by statutory health insurance in Germany (“Gesetzliche Krankenversicherung [GKV]) available for the period 2004 to 2014 was analyzed. The sociodemographic structure of the population included (5.2 million) is considered widely representative of the entire GKV population in Germany (80 million). The following databases were used: registration data (demography, time insured, and regional distribution), outpatient and inpatient cares, drug prescriptions, sick leaves, and sick benefits data. Using a person‐related pseudonym, it was possible to unambiguously identify patients in all datasets abovementioned.

### 2.5 Statistical methods

For the primary outcome, the 3 cohorts (OM, other ARBs, or ACE‐i) were stratified according to the incidence of the events, calculated as the number of hospitalizations for unspecified IM per person‐years (PY). Furthermore, a modified Poisson regression model adjusted for the following potential confounders was used: age, sex, and presence of at least 1 comorbidity among those of interest (diabetes, transplantation, malignant neoplasms, and renal failure). A modified Poisson regression was performed by using a robust error variance procedure (known as *sandwich* estimation) that is used to analyze correlated data, which can occur because of clustered data.^30^ Failure to account for the correlation in the data can result in underestimating the variance, which would lead to artificially low *P* values.^31^ In the present context, this approach was used to account for the different clusters (LHUs and countries) and to correctly estimate the standard error for the estimated relative risk. The same methodology was applied for the secondary outcome stratifying the 3 cohorts according to the incidence of the events calculated as the number of hospitalizations for IM (Germany: K90.x; Italy: 579.x) per PY.

## 3 RESULTS

The final cohort included 465.443 patients divided into 3 groups of treatment: OM (25.591 patients, 5.5%), other ARBs (104.901 patients, 22.5%), and ACE‐i (334.951 patients, 72.0%). Baseline characteristics showed little differences among treatments groups (Table 1). Specifically, OM and ACE‐i patients were slightly younger when compared with the other ARB group. Women were overrepresented (52.1%) in the other ARB group compared with the OM (49.7%) or ACE‐i (46.3%) groups. The OM group contributed with the lowest percentage of patients with at least 1 of the comorbidities of interest. Crude incidence rates of events on total PY of exposure to treatments are presented in Table 2. Regarding the primary outcome, 23 hospitalizations for unspecified IM were observed, 12 in the other ARB group, 10 in the ACE‐i group, and 1 in the OM group, yielding crude incidence rate of 8.8 per 100.000 PY, 2.3 per 100.000 PY, and 3.1 per 100.000 PY, respectively. Table S2 reports the crude incidence rates of events by treatment groups defined as ARBs (including OM) or ACE‐i.

**Table 1:** Population characteristics at baseline

Demographic Characteristics	Olmesartan	Other ARBs	ACE‐i
Patients (n, %)	25,591 (5.5)	104,901 (22.5)	334,951 (72.0)
Age (mean, SD)	63.0 (14.0)	65.0 (14.4)	62.4 (14.7)
Age (n, %)			
Missing	17 (0.1)	143 (0.1)	862 (0.3)
<50	4,757 (18.6)	16,803 (16.1)	68,349 (20.4)
50–60	5,916 (23.1)	20,826 (19.9)	77,950 (23.3)
60–70	5,923 (23.1)	22,839 (21.8)	70,655 (21.1)
70–80	5,575 (21.8)	25,626 (24.4)	71,065 (21.2)
≥80	3,403 (13.3)	18,664 (17.8)	46,070 (13.8)
Female (n, %)	12,728 (49.7)	54,597 (52.1)	154,923 (46.3)
Comorbidities^a^	3,789 (14.8)	21,982 (21.0)	87,636 (26.1)
Diabetes (n, %)	2,301 (9.0)	12,190 (11.6)	46,899 (14.0)
Transplantation (n, %)	35 (0.1)	285 (0.3)	959 (0.3)
Malignant neoplasms (n, %)	1,021 (4.0)	6,931 (6.6)	32,300 (9.6)
Renal failure (n, %)	432 (1.7)	2,576 (2.5)	7,478 (2.2)

ACE‐i indicates angiotensin converting enzyme inhibitor; ARB, angiotensin II receptor blocker.

a The total number of comorbidities could exceed the total number of patients with at least 1 comorbidity because 1 patient could have more than 1 comorbidity of interest.

**Table 2:** Number and crude incidence rates of unspecified intestinal malabsorption and intestinal malabsorption events (Germany: ICD10: K90.4. K90.8. K90.9; Italy: ICD‐9 codes: 579.8 or 579.9) stratified by treatment at index date

Outcome	Index Date Treatment	Number of Events	Person‐Year (PY)	Unadjusted Incidence Rate *100,000 PY	Lower 95% Confidence Limit	Upper 95% Confidence Limit
Unspecified intestinal Malabsorption^a^	Olmesartan	1	32,041	3.12	0.44	22.16
	Other ARBs	12	136,827	8.77	4.98	15.44
	ACE‐i	10	431,139	2.32	1.25	4.31
Intestinal malabsorption^b^	Olmesartan	4	32,035	12.49	4.69	33.27
	Other ARBs	20	136,818	14.62	9.43	22.66
	ACE‐i	39	431,123	9.05	6.61	12.38

ACE‐i indicates angiotensin converting enzyme inhibitor; ARB, angiotensin II receptor blocker.

a ICD‐10: K90.4, K90.8, K90.9; ICD‐9:579.8, 579.9.

b ICD‐10: K90.x; ICD‐9: 579.x.

When we looked at the primary outcome, the risk of hospitalization of ACE‐i patients compared with OM was not significantly different in both crude and adjusted analyses (Table 3). On the other hand, adjusted rate ratios (RRs) showed that other ARB users were associated with an over 2‐fold significantly higher risk of hospitalization compared with OM (RR: 2.50, 95% CI 1.21‐5.19, *P* .01, Table 3). In addition, age was a significant covariate in the Poisson‐modified model: Modestly higher IM risk (RR = 1.03) was significantly associated for each year‐unit increase of age (*P* = .02). When we looked at the secondary outcome of the analysis, there were 63 hospitalizations for IM, 20 of which related to the other ARB group, 39 to the ACE‐i group, and 4 to the OM group, resulting in a crude incidence rate of 14.6 per 100.000 PY, 9.1 per 100.000 PY, and 12.5 per 100.000 PY, respectively. Table S3 reports the distribution of events by discharge diagnosis code. In the overall analysis comprising all ICD codes, no significant differences in risk of hospitalization were measured among treatments (Table 3). In particular, other ARBs and ACE‐i reported an adjusted RR of, respectively, 1.06 and 0.73 of hospitalizations compared with OM, and both estimates were nonsignificant. Women showed a significantly higher risk than men (RR = 1.68, *P* value: .01). Neither age nor the presence of at least 1 comorbidity had influence on the RRs.

**Table 3:** Crude and adjusted rate ratios of hospitalization with a discharge diagnosis of unspecified intestinal malabsorption (Germany: ICD10: K90.4. K90.8. K90.9; Italy: ICD‐9 codes: 579.8 or 579.9) and intestinal malabsorption (Germany: ICD‐10 codes K90x. Italy: ICD‐9 codes 579×) and 95% CI (ref: Olmesartan)

Outcome	Parameter	Crude RR	95% CI	*P* Value	Adjusted RR	95% CI	*P* Value
Unspecified intestinal malabsorption^a^	Other ARBs	2.93	(0.35 to 24.55)	.3224	2.50	(1.21 to 5.19)	.0134
	ACE‐i	0.76	(0.11 to 5.13)	.7773	0.78	(0.19 to 3.19)	.7255
	Female	—	—	—	0.89	(0.48 to 1.64)	.7008
	Comorbidity^c^	—	—	—	0.78	(0.24 to 2.58)	.6891
	Age	—	—	—	1.03	(1.01 to 1.06)	.0171
Intestinal malabsorption^b^	Other ARBs	1.16	(0.64 to 2.12)	.6190	1.06	(0.58 to 1.96)	.8420
	ACE‐i	0.73	(0.34 to 1.55)	.4078	0.73	(0.37 to 1.46)	.3736
	Female	—	—	—	1.68	(1.15 to 2.44)	.0069
	Comorbidity^c^	—	—	—	1.43	(0.81 to 2.51)	.2204
	Age	—	—	—	1.00	(0.99 to 1.01)	.3709

ACE‐i indicates angiotensin converting enzyme inhibitor; ARB, angiotensin II receptor blocker; CI, confidence interval; RR, rate ratio.

a ICD‐10: K90.4, K90.8, K90.9; ICD‐9:579.8, 579.9.

b ICD‐10: K90.x; ICD‐9: 579.x.

c At least 1 comorbidity among those of interest.

## 4 DISCUSSION

In the pooled analysis of 2 large cohorts of patients obtained from Italian administrative and German statutory health insurance (GKV) databases, we reported a low incidence rate of IM hospitalizations for patients on treatment with ARBs and ACE‐i. In addition, we found that drug‐associated sprue, codified as unspecified IM diagnoses, was more common in the group of ARB patients when adjusting for possible confounders. At the same time, we could not confirm previous findings of a higher risk of malabsorption in olmesartan‐only patients. The presence of an association between ARBs and malabsorption that emerges from the present study is in line with previously conducted studies and strengthens the class‐effect hypothesis recently suggested elsewhere.^10^, ^13^, ^14^, ^15^^,^ 21 Despite the large number of patients in the study, only 1 episode of malabsorption‐related hospitalization was detected in the group of OM patients. This result is in line with indications from previous RCT and case‐control studies that reported no differences in the occurrence of diarrhea, abdominal discomfort, and diagnosis of coeliac disease or microscopic colitis when comparing olmesartan with control groups.^32^, ^33^ However, this result is in contrast with the findings by Basson et al, who reported a higher risk of malabsorption in patients treated with OM, even if confirming that olmesartan‐induced enteropathy remains a rare condition.^11^ A possible explanation of the difference between the 2 studies is that, in the present study, the primary outcome was defined by using ICD codes for *unspecified* IM based on the assumption that a clinician, in the absence of a specific code defining SLE diagnosis, would select a general and nonspecific diagnosis code. In fact, in the absence of a definitive etiology for villous atrophy, patients are most likely characterized as having unclassified sprue, a diagnosis of exclusion, for which the optimal management is still unknown.^8^ Nevertheless, because SLE is an adverse drug reaction that mimics the appearance of celiac disease, in this study, we also assessed the risk of IM considering all ICD‐10K90.x and ICD‐9 579.x codes for Germany and Italy, respectively. We found crude incidence rates of malabsorption in our 2 cohorts almost 4‐fold higher for ACE‐i, 8‐fold higher for other ARBs, and 2‐fold higher for OM‐treated patients when compared with the findings retrieved in similar cohorts in France.^11^ Moreover, a significant higher risk in women than in men was found, which is probably because about 42% of the events were hospitalizations for coeliac disease, which is well known to predominantly characterize the female gender. While inclusion criteria were similar to the French study, exclusion criteria were different due to limited availability of some information in the German and Italian databases, and this may well account for the difference in magnitude in the incidence rates. In particular, in the study by Basson et al, all patients with any prescriptions for gluten‐free diet before the index date were excluded. In the present analysis, it was not possible to retrieve the latter information; therefore, hospitalizations for celiac disease (representing over 42% of total events in our study) could include those patients already carrying celiac disease before the index date. This could partly explain the increased total number of malabsorption‐related hospitalizations, although affecting all studied drugs to a similar extent. Another factor potentially driving up the hospitalizations number is that the present study ended later (ie, 2015 Italy and 2014 Germany) than the French study (2012). Given that Olmesartan‐induced SLE was first reported in 2012,^6^ it is possible to hypothesize that this could have led to an increased awareness of physicians about the existence of a drug‐associated enteropathy in the subsequent years. The study presents the following limitations. First, treatment exposure is based on prescribed and dispensed prescriptions by pharmacies, and no information on actual use of the drugs is available. Second, we used administrative databases with limited clinical information available, which would have been helpful to better characterize the drug‐associated malabsorption events. Although this represents a standard approach in real‐world evidence studies, severity, clinical factors, and therapeutic patterns were assessed by means of surrogate tools (ie, hospitalization, examinations, exemptions, and prescribed drugs). Third, our study refers to the more serious cases of IM, ie, patients who required hospitalization. In analogy to Basson et al, the aim of our study was to assess the prevalence in the association between ARBs and ACE‐I treatments for severe forms of enteropathy. Cases with mild forms of malabsorption as observed in outpatient setting were not included in the study. This study has also several strengths. To minimize the bias effect of concomitant conditions, analyses were adjusted for confounders (diabetes, cancer, renal failure, and transplantation) potentially influencing the risk of developing severe enteropathy. Another strength of the present study was the exclusion of patients with hospitalizations for IM and coeliac disease as well as patients with any diagnosis of malabsorption before the index date. Also, the use of national representative databases designed for administrative and billing purposes provides insight into otherwise difficult‐to‐study, low‐incidence clinical events and outcomes.^34^

In conclusion, this study suggests that drug‐induced enteropathy should be considered the result of exposure to the class of ARBs rather than a specific drug‐related effect. Yet, the higher risk found in relation to the ARBs users is sensitive to the ICD codes considered in our study. Similarly, it has to be stressed that to better understand the causality of ARB‐associated sprue and its magnitude, other studies accounting for different populations, different study designs, different time periods, and duration of drug exposure alongside the consideration of specific population‐related lifestyles, individual susceptibility, and co‐administered drugs should be encouraged.^35^ Mechanisms of drug‐induced enteropathy await further exploration.

## ETHICS STATEMENT

Approval by the pertinent Ethical Committees and Competent Authorities were obtained in accordance with all the regulations in force and regulatory requirements.

## CONFLICT OF INTEREST

Nazarena Cataldo is an employee of QuintilesIMS. Claudio Ripellino has received research funding from QuintilesIMS. Peter Malfertheiner has received funding from Menarini Industrie Farmaceutiche Riunite Srl.

## AUTHOR'S CONTRIBUTION

Nazarena Cataldo is acting as the submission's guarantor and takes responsibility for the integrity of the work as a whole, from inception to published article. Prof. Peter Malfertheiner was responsible for clinical validation of the study and review of the manuscript. Claudio Ripellino has served as an RWE HEOR consultant for QuintilesIMS. Claudio Ripellino and Nazarena Cataldo designed the research study, performed the research, collected and analyzed the data, and wrote the paper. All authors read and approved the final manuscript.

## Supporting information

Table S1. Selection period per LHU
Table S2. Number and crude incidence rates of unspecified intestinal malabsorption and intestinal malabsorption events (Germany: ICD10: K90.4. K90.8. K90.9; Italy: ICD‐9 codes: 579.8 or 579.9) stratified by treatment groups
Table S3. Number of intestinal malabsorption events stratified by treatment at index date and diagnosis code typology

## References

[1] Freeman HJ . Sprue‐like intestinal disease. International Journal of Celiac Disease. 2014;2(1):6‐10.

[2] Adike A , Corral J , Rybnicek D , Sussman D , Shah S , Quigley E . Olmesartan‐induced enteropathy. Methodist DeBakey Cardiovascular Journal: December 2016. 2016;12(4):230‐232. 10.14797/mdcj-12-4-230 PMC5344475 28289500

[3] Scialom S , Malamut G , Meresse B , et al. Gastrointestinal disorder associated with Olmesartan mimics autoimmune enteropathy. PLoS ONE. 2015;10(6). 10.1371/journal.pone.0125024 PMC4477936 26101883

[4] Ianiro G , Bibbò S , Montalto M , Ricci R , Gasbarrini A , Cammarota G . Systematic review: sprue‐like enteropathy associated with olmesartan. Aliment Pharmacol Ther. 2014;40(1):16‐23. 24805127 10.1111/apt.12780

[5] Freeman HJ . Drug‐induced sprue‐like intestinal disease. International Journal of Celiac Disease. 2014;2(2):49‐53.

[6] Rubio‐Tapia A , Herman ML , Ludvigsson JF , et al. Severe spruelike enteropathy associated with Olmesartan. Mayo Clin Proc. 2012;87(8):732‐738. 22728033 10.1016/j.mayocp.2012.06.003 PMC3538487

[7] Fiorucci G , Puxeddu E , Colella R , Paolo Reboldi G , Villanacci V , Bassotti G . "Severe spruelike enteropathy due to olmesartan," 2014. 10.4321/s1130-01082014000200011 24852741

[8] DeGaetani M , Tennyson CA , Lebwohl B , et al. Villous atrophy and negative celiac serology: a diagnostic and therapeutic dilemma. Am J Gastroenterol. 2013;108(5):647‐653. 23644957 10.1038/ajg.2013.45

[9] Dreifuss SE , Tomizawa Y , Farber NJ , Davison JM , Sohnen AE . Spruelike enteropathy associated with olmesartan: an unusual case of severe diarrhea. Case Rep Gastrointest Med. Mar 2013. 10.1155/2013/618071 PMC3610358 23573432

[10] Marthey L , Cadiot G , Seksik P , et al. Olmesartan‐associated enteropathy: results of a national survey. Aliment Pharmacol Ther. 2014;40(9):1103‐1109. 25199794 10.1111/apt.12937

[11] Basson M , Mezzarobba M , Weill A , et al. Severe intestinal malabsorption associated with olmesartan: a French nationwide observational cohort study. Gut. 2016;65(10):1664‐1669. 26250345 10.1136/gutjnl-2015-309690

[12] Zanelli M , Negro A , Santi R , et al. Letter: sprue‐like enteropathy associated with angiotensin II receptor blockers other than olmesartan. Aliment Pharmacol Ther. 2017;46:471‐473. 28707802 10.1111/apt.14176

[13] Mondet L , Pierson‐Marchandise M , Gras V , et al. Angiotensin II receptor blockers‐induced enteropathy: not just olmesartan! A case report with candesartan. Fund Clin Pharmacol. 2016;30:62.

[14] Maier H , Hehemann K , Vieth M . Celiac disease‐like enteropathy due to antihypertensive therapy with the angiotensin‐II receptor type 1 inhibitor eprosartan. Cesk Patol. 2015;51(2):87‐88. 25970720

[15] Cammarota G , Ianiro G , Bibbò S , Gasbarrini A . Letter: telmisartan associated enteropathy—is there any class effect? Authors' reply. Aliment Pharmacol Ther. 2014;40(5):570. 25103354 10.1111/apt.12870

[16] Negro A , Rossi GM , Santi R , Iori V , De Marco L . A case of severe sprue‐like enteropathy associated with losartan. J Clin Gastroenterol. 2015;49(9):794. 10.1097/MCG.0000000000000383 26166143

[17] Herman ML , Rubio‐Tapia A , Wu TT , Murray JA . A case of severe sprue‐like enteropathy associated with valsartan. ACG Case Rep J. 2015;2(2):92‐94. 26157924 10.14309/crj.2015.15 PMC4435362

[18] Sawant AP , Spoletini G , Etherington C , et al. Losartan‐associated sprue‐like enteropathy in a post‐lung transplant cystic fibrosis patient. J Cyst Fibros. 2017;16:S135.

[19] Cyrany J , Vasatko T , Machac J , Nova M , Szanyi J , Kopacova M . Letter: telmisartan associated enteropathy—is there any class effect? Aliment Pharmacol Ther. 2014;40:569‐570. 25103353 10.1111/apt.12850

[20] Mandavdhare HS , Sharma V , Prasad KK , Kumar A , Rathi M , Rana SS . Telmisartan‐induced sprue‐like enteropathy: a case report and a review of patients using non‐olmesartan angiotensin receptor blockers. Intest Res. 2017;15(3):419‐421. 28670240 10.5217/ir.2017.15.3.419 PMC5478768

[21] De Bortoli N , Ripellino C , Cataldo N , Marchi S . Unspecified intestinal malabsorption in patients treated with angiotensin‐converting enzyme inhibitors or angiotensin receptor blockers: a retrospective analysis in primary care settings. Expert Opin Drug Saf. 2017;16(11):1221‐1225. 28871813 10.1080/14740338.2017.1376647

[22] Ruggeri I , Bragato D , Colombo GL , Valla E , Di Matteo S . Cost and appropriateness of treating asthma with fixed‐combination drugs in local healthcare units in Italy. Clinicoecon Outcomes Res. 2012;4:375‐382. 23233808 10.2147/CEOR.S36499 PMC3519004

[23] Colombo GL , Rossi E , De Rosa M , Benedetto D , Gaddi AV . Antidiabetic therapy in real practice: indicators for adherence and treatment cost. Patient Prefer Adherence. 2012;6:653‐661. 23055698 10.2147/PPA.S33968 PMC3461605

[24] Buffa S , Camrda M , Cananzi P et al. "Report analisi data base amministrativi Regione Sicilia—studio trilogy," Il Pensiero Scientifico Editore, vol. Supplemento n 1, 2012.

[25] Delsole P , Gioia AF , Bolzoni E , "L'utilizzo di database amministrativi per l'analisi della gestione della BPCO: il caso dell'inappropriatezza prescrittiva di farmaci a combinazione fissa (FDC)," Il Pensiero Scientifico Editore, vol. Supplemento n. 1, 2011.

[26] Santoni L , Dall'Asta G , Spampinato A , et al. Aderenza e persistenza alla terapia con statine: analisi di farmacoutilizzazione a partire dai database amministrativi di cinque ASL italiane. Giornale Italiano di Farmacoeconomia e Farmacoutilizzazione. 2009;2(1):5‐16.

[27] Sangiorgi D , Perrone V , Buda S , et al. Epidemiology, patient profile, and health care resource use for hepatitis C in Italy. Clinicoecon Outcomes Res. 2017;9:609‐616. 29066922 10.2147/CEOR.S136456 PMC5644550

[28] Banks H , Torbica A , Valzania C , et al. Five year trends (2008‐2012) in cardiac implantable electrical device utilization in five European nations: a case study in cross‐country comparisons using administrative databases. Europace. 2017. 10.1093/europace/eux123 29016747

[29] Carnevale V , Fontana A , Scillitani A , Sinisi R , Romagnoli E , Copetti M . Incidence and all‐cause mortality for hip fracture in comparison to stroke, and myocardial infarction: a fifteen years population‐based longitudinal study. Endocrine. 2017. 10.1007/s12020-017-1423-1 28933053

[30] Zou G . A modified poisson regression approach to prospective studies with binary data. Am J Epidemiol. 2004;159(7):702‐706. 15033648 10.1093/aje/kwh090

[31] Orelien JG , "Model fitting in PROC GENMOD.," in 26th Annual SAS Users Group International Conference, 2001, pp. 22–25.

[32] Greywoode R , Braunstein ED , Arguelles‐Grande C , Green PH , Lebwohl B . Olmesartan, other antihypertensives and chronic diarrhea among patients undergoing endoscopic procedures: a case‐control study. Mayo Clinic Proc. 2014;89(9):1239‐1243. 10.1016/j.mayocp.2014.05.012 PMC4157109 25023670

[33] Menne J , Haller H . Olmesartan and intestinal adverse effects in the ROADMAP study. Mayo Clin Proc. 2012;87(12):1230‐1232. 23218089 10.1016/j.mayocp.2012.10.005 PMC3547579

[34] Memtsoudis SG . Limitations associated with the analysis of data from administrative databases. Anesthes. 2009;111(2):449. author reply 450‐451 10.1097/ALN.0b013e3181adf739 19625813

[35] Hill AB , "The environment and disease: association or causation? ," pp. 295–300, 1965. 10.1177/003591576505800503 PMC1898525 14283879

