# Supplementary material for: Severe intestinal malabsorption associated with ACE inhibitor or angiotensin receptor blocker treatment. An observational cohort study in Germany and Italy
Source: Pharmacoepidemiol Drug Saf. 2018 May 1;27(6):581–6. doi: 10.1002/pds.4402 (PMC6001476; doi:10.1002/pds.4402)
Supplement: Supplementary file 1 — Table S1. Selection period per LHU Table S2. Number and crude incidence rates of unspecified intestinal malabsorption and intestinal malabsorption events (Germany: ICD10: K90.4. K90.8. K90.9; Italy: ICD‐9 codes: 579.8 or 579.9) stratified by treatment groups Table S3. Number of intestinal malabsorption events stratified by treatment at index date and diagnosis code typology [file PDS-27-581-s001.docx]

**Supporting information**

Table S1: Selection period per LHU

| Region | Public corporation | Selection period |
| --- | --- | --- |
| LOMBARDIA - LAZIO | ATS di Bergamo - ASL Roma3 | Jan 2011 – Dec 2015 |
| SICILIA | Asp Palermo | Jan 2013 – Dec 2015 |
| TOSCANA - BASILICATA | Azienda Usl Toscana sud est -  Azienda Sanitaria Locale di Matera ASM | Jan 2014 – Dec 2015 |

### Table S2: Number and crude incidence rates of unspecified intestinal malabsorption and intestinal malabsorption events (Germany: ICD10: K90.4. K90.8. K90.9; Italy: ICD-9 codes: 579.8 or 579.9) stratified by treatment groups

| Outcome | Index Date Treatment | Number of events | Person-Year | Unadjusted Incidence Rate *100000 PY | Lower 95% Confidence Limit | Upper 95% Confidence Limit |
| --- | --- | --- | --- | --- | --- | --- |
| **Unspecified Intestinal Malabsorption^1^** | **ARBs** | 13 | 168868 | 7.69 | 4.09 | 13.16 |
|  | **ACE-i** | 10 | 431139 | 2.32 | 1.11 | 4.27 |
| **Intestinal Malabsorption^2^** | **ARBs** | 24 | 168853 | 14.21 | 9.10 | 21.15 |
|  | **ACE-i** | 39 | 431123 | 9.05 | 6.43 | 12.37 |

^1^ ICD-10: K90.4, K90.8, K90.9; ICD-9:579.8, 579.9

^2^ ICD-10: K90.x; ICD-9: 579.x

Table S3– Number of intestinal malabsorption events stratified by treatment at index date and diagnosis code typology

| ICD codes | Olmesartan | Other ARBs | ACE-i |
| --- | --- | --- | --- |
| K90.0  579.0 | 3 | 5 | 19 |
| K90.1. K90.2. K91.2. K90.3  579.1. 579.2. 579.3. 579.4 | - | 4 | 10 |
| K90.4. K90.8. K90.9  579.8. 579.9 | 1 | 12 | 10 |
| Total | **4** | **21** | **39** |

The total numbers of events per treatment (column other ARBs) exceeds the total number of events reported since one patient had one hospitalization related to two different ICD codes. Anyway. in the calculation of crude incidence rates this patient accounted for one event.
